# The Relationship between Landscape Construction and Bird Diversity: A Bibliometric Analysis

**DOI:** 10.3390/ijerph20054551

**Published:** 2023-03-03

**Authors:** Yanqin Zhang, Enming Ye, Fan Liu, Ningjing Lai, Xianli You, Jianwen Dong, Jiaying Dong

**Affiliations:** 1 College of Landscape Architecture and Art, Fujian Agriculture and Forestry University, Fuzhou 350002, China; 2 Engineering Research Center for Forest Park of National Forestry and Grassland Administration, Fuzhou 350002, China; 3 School of Architecture, Clemson University, Clemson, SC 29634, USA

**Keywords:** landscape planning and design, bird habitat, urban green, urbanization, CiteSpace, knowledge map

## Abstract

Urbanization development is the main cause of drastic habitat changes and biodiversity loss, and urban green space construction is one of the effective ways to mitigate biodiversity decay. The proper construction of urban green space landscapes can maintain or increase the resources provided by urban biodiversity, especially bird diversity. This paper is based on 4112 papers published in this research area between 2002 and 2022, and CiteSpace was used to conduct a bibliometric analysis of the research area in terms of the number of articles published, the country or region of publication, core authors, and academic development. The paper systematically reviews the hotspots, history, and frontiers of research on landscape architecture and bird diversity. At the same time, the relationship between landscape construction and bird diversity is discussed in the context of landscape features, vegetation characteristics, and human behavioral activities. The results revealed: (1) research on the association between landscape camping and bird diversity received high priority from 2002 to 2022. Moreover, this research area has become a mature discipline. (2) Throughout the research history, there are four research hotspots (fundamental research on bird communities, influencing factors related to changes in bird community characteristics, research on bird activity rhythms, and ecological and ornamental values of birds), four development stages (2002–2004, 2005–2009, 2010–2015, and 2016–2022), and several research frontiers. (3) Our aim was to reasonably consider the activity characteristics of birds in future landscape construction, and to thoroughly study the landscape construction strategies and management principles for the harmonious coexistence of humans and birds.

## 1. Introduction

The continued advance of urbanization has been identified as a major cause of habitat destruction and biodiversity loss [1,2]. As cities continue to expand, especially in biodiversity rich areas, urbanization will pose a serious threat to global biodiversity [3]. The loss of biodiversity due to urbanization is a serious issue of global concern [4]. Urbanization often leads to severe changes in the diversity and distribution of many species and affects the quality and quantity of ecosystem services available to people living in urban areas [5,6]. Global biodiversity is declining at a much faster rate than previously anticipated, and action must be taken to balance the relationship between people and nature. Development minimizes ecological loss and maximizes human wellbeing. Since the ratification of the Convention on Biological Diversity, local readjustment programs have become the primary tool used in many countries to manage biodiversity, promote local action, and inform overall urban planning and decision making [7].

In this context, green spaces are important for the conservation of urban biodiversity. Urban green spaces provide habitat for wildlife and increase the functional connectivity of local fauna [8]. At the same time, parks in urban areas are also “islands” or habitat fragments for wildlife [9]. At the same time, the construction of urban animal habitats is closely related to urban landscape construction. For example, urban green areas, parks, communities, tree corridors, gardens, etc. are important habitats for various bird species in cities [10]. Therefore, the urban landscape is closely related to wildlife survival. The factors influencing the level of landscape also affect the bird communities within cities [11]. Urban landscapes, including green spaces and park landscapes, are inhabited by a variety of native birds, which help maintain biodiversity in urban landscapes. However, the surrounding landscape affects their ability to support native birds and protect urban biodiversity [12].

As an important component of biodiversity, birds are an important indicator of the health of urban ecosystems [13]. Moreover, birds are an important part of the natural ecosystem and an important part of the food chain. Once the ecosystem is disturbed, it will have a great impact on birds. Birds are more sensitive to environmental changes, indicative of environmental changes, more numerous, and bird communities are easier to observe [14]. Birds are often used to evaluate environmental strengths and weaknesses and are often used to monitor ecosystems by studying bird communities. They are one of the indicators that indirectly respond to the state of the ecosystem. Therefore, there is a growing interest in studying the relationship between landscape characteristics and bird communities by observing the characteristics, status, and behavior of bird communities.

Urban landscape creation and bird diversity are correlated. Urban green space area has a positive effect on bird species richness [15]. Urban park green space size is an important factor affecting bird abundance and diversity [16]. Urban park area and bird diversity showed a positive correlation [17]. In addition, urban landscape fragmentation, connectivity, and urban rural gradients also have an impact on bird pair positivity. Meanwhile, the vegetation composition and structure of urban parks have an influence on birds, and horizontal vegetation cover is particularly important for birds [18,19]. In addition, the presence of humans is also generally considered to have a negative impact on the richness and diversity of bird species [20,21]. These suggest that urban green space landscaping needs to consider both human and bird habitats. Therefore, it is important to explore the influencing factors of bird diversity at the landscape level and to discuss how to build the landscape from the perspective of bird diversity in order to improve the habitat and enhance urban biodiversity. 

The study used a comprehensive bibliometric analysis to explore the patterns and development history of research between landscape camping and bird diversity. This study analyzed the number of published articles, countries or regions of publication, core authors, and academic developments. This was followed by keyword analysis (research hotspots, research history, and research frontiers), combined with visual mapping drawn by bibliometric software to provide a systematic and detailed description of the intellectual background of the research area. The relationship that exists between landscape camping and bird diversity is also discussed from three aspects (landscape characteristics, vegetation characteristics, and human behavioral activities). It will help scholars to value the role of landscape architecture on urban bird diversity and to better understand the overview and recent progress in the field. These studies were retrieved from the Web of Science (WoS) core collection database and analyzed using CiteSpace visualization software to create a visual knowledge network with a focus on four key points:

1. What are the current and growing trends in publishing in the field of landscape construction and bird diversity research?
2. What countries/regions and authors have influenced landscape construction and bird diversity research?
3. What are the research hotspots and historical and frontier issues in the field of landscape construction and bird diversity research?
4. What relationships exist between landscape features, vegetation features and human behavioral activities and bird diversity?

In this study, this paper assesses the literature and its articles in the field of landscape camping and bird diversity research published between 2002 and 2022, and then provides a more precise and specific analysis based on a collaborative network map of countries/regions and authors in the research field. This study also includes a complete analysis and description of keywords (distribution, history, and frontiers) to better reflect research hotspots and directions, as well as the interactions between research hotspots and time scales. Based on this, the paper assesses the relationship between landscape camping and bird diversity in three ways. Finally, this paper proposes a research direction for the sustainable development of urban bird diversity through landscape architecture.

## 2. Materials and Methods

### 2.1. Data Collection

On 24 February 2023, 4661 articles were retrieved from the Web of Science (WoS) core collection database. This WoS core collection database was chosen for the collection of the research literature for this study. The search formula was (TS = (landscape OR park OR green land OR plant OR bird habitat) AND TS = (bird Diversity) AND TS = (design* OR plan* OR build* OR construct*)) AND ((LA == (“ENGLISH”)) NOT (PY == (“2023”))). In this paper, the literature was imported into CiteSpace for deduplication. Article types were selected for article and review, and after screening and review 4549 articles were finally used for analysis.

### 2.2. Data Analysis

The bibliometric is a set of quantitative tools for analyzing bibliographic data. These tools can fully analyze information related to the published literature in the field of study, including publications (year, country or region, author, etc.), keywords, and citation trend analysis, to help scholars fully grasp the knowledge structure of the field of study [22].

Visualization and analysis software frequently used includes CiteSpace, VOSviewer, Histsite [23], Bibexcel [24], and R-Package Bibliography [25]. In particular, CiteSpace supports many types of bibliometric studies, including collaborative network analysis, co-word analysis, author co-citation analysis, document co-citation analysis, and textual and geospatial visualization, and its bibliographic and visualization functions can present the trends and knowledge association status of disciplinary frontiers in a very visual way, which can allow the key information of the research field to be grasped quickly [26,27,28]. Therefore, this paper uses CiteSpace (5.7.R5) to quantitatively evaluate the relevant literature (number of articles issued, country region of issue, core authors, academic development). In addition, we constructed corresponding knowledge maps (keyword co-occurrence map, time zone map, and highlight map) based on the literature in the field of landscape architecture and bird diversity research, and identified research hotspots, history, and frontiers, in order. We also explore the relationship between landscape architecture and bird diversity from three aspects of landscape architecture (landscape characteristics, vegetation characteristics, and human behavioral activities). The purpose is to provide a reference and foundation for future research.

## 3. Basic Situation Analysis

### 3.1. Trends in the Number of Published Papers

**Figure 1:**
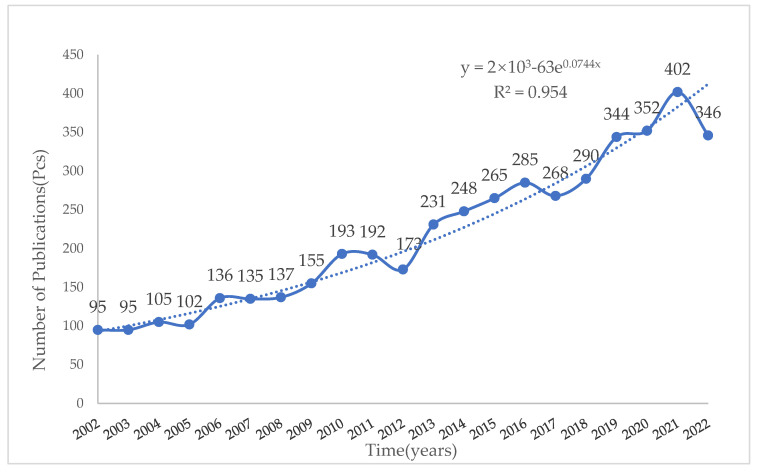
Statistical chart of the number of articles issued.

By analyzing the annual changes in the number of publications in a research field, it is possible to assess the current state of research in the field and to predict future trends. In this paper, we analyzed a total of 4549 publications issued between 2002 and 2022 to obtain the number of publications per year for the last two decades and to forecast their trends (Figure 1). The field of landscape camping and bird diversity research has steadily developed over the last two decades. Figure 1 shows that from 2002 to 2022 the number of studies in the subject area of the scientific network database increased year by year, and the research history can be divided into three phases: (1) the initial phase, from 2002 to 2005, with the number of articles between 95 and 105 per year; (2) the cumulative phase from 2006 to 2012, with the number of publications remaining relatively stable each year, with the number of articles between 130 and 200 per year, with 2012 being the inflection point for development; and (3) a steady growth phase from 2013 to 2022, with the volume of the literature steadily increasing year by year and fluctuating during 2017–2018, but remaining at 250 to 300 articles per year. The number of publications increased by 402 articles in 2021.

The growth trend of the field of study is predicted by compiling year-by-year statistics of the number of articles published in the field of study and by means of a Price’s curve. The growth of the scientific literature is exponentially related to time, and the closer the coefficient of determination of the trend line is to 1, the better the fit is. It also indicates the faster growth rate of the number of publications in a given subject area in the future [29] (Figure 1). Figure 1 shows that the sample paper load versus time is given by the equation y = 2×10^3^-63e^0.0744x^, and its trend line determinant is R^2^ = 0.954. It reveals that the number of publications in this research area show a clear exponential growth trend as time advances. The exponential line of publications in recent years, with an R-squared value of R^2^ = 0.954, represents its reliability. The closer the R-squared value is to 1, the better the fit of the exponential line to the figures. This demonstrates the reliability of the equation in predicting the number of articles published in this research area. Moreover, it indicates that the development of the field of landscape camping and bird diversity research is accelerating.

### 3.2. Country/Region Cooperation Networks

By analyzing the collaborative networks between countries/regions, it is possible to identify priority countries/regions that produce a large number of publications and have a significant impact on the research field, and to determine the collaborative relationships between them [30]. Academic articles in the field of landscape architecture and bird diversity have been published in 134 countries/regions. Centrality indicates the importance of a particular node in the network. Therefore, the greater the centrality, the greater the influence of the posting in that country/region [31]. A list of the top 20 countries with the largest number and impact of publications in this research area can be found in Table 1. Most countries worldwide are involved in this field of study, with the USA being the most published country in this field (1311), followed by England (488), Germany (410), and Australia (387), among others. In terms of centrality, England (0.28) has the highest score, followed by Australia (0.1), France (0.1), and the USA (0.08); although the number of articles published in England, Australia and France is less than that of the USA, their impact is higher than that of the USA.

The USA is dedicated to studying the effects of urbanization, habitat structure changes, and human drivers on birds, and the effects of urban landscape vegetation on birds [6,32,33,34]. In addition, the UK has focused on the impact of different environmental schemes on biodiversity [35,36], with an emphasis on the ecological impacts of changes in agricultural landscapes [37,38]. Moreover, the biodiversity values of forest, secondary forest and planted forest are compared [39]. Germany focuses on the effects of landscape structure and land use intensity on plant, bird and other flora and fauna communities [40]. Furthermore, the differences in biodiversity between managed and non-managed forests and the interrelationship between biodiversity and land use intensity are investigated [41,42]. Australia focuses on the relationship between urban–rural gradients and biodiversity [43]. The French studies include the effects of climate change on the phylogenetic diversity of plant, bird, and mammalian communities across Europe [44]. China ranks sixth in the number of articles published in this field, but its influence is not high and still needs further development.

### 3.3. Author Cooperation Networks

**Figure 2:**
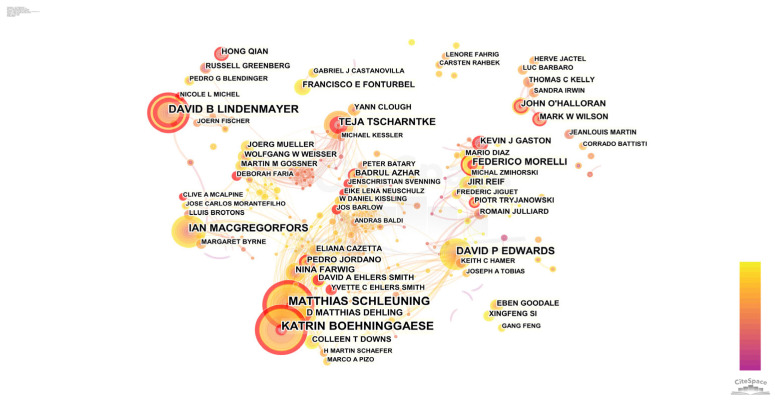
Author collaboration network map.

It is useful to know how scholars interact in the research field to understand the research dynamics of the research area. A total of 4549 studies were searched for in CiteSpace, and the authors were obtained with the time span from 2002 to 2022, node type “author”, and parameters set to the software default parameters. The collaborative network mapping is shown in Figure 2 and the table of the top ten authors in terms of number of publications and centrality is in Table 2. The base information of the graph is N = 830, E = 1394, and Density = 0.0041. The size of the nodes in the graph reflects the number of articles published by scholars, and the node linkage and the thickness of the line segments reflect the cooperation relationship and the frequency of cooperation among scholars. From Figure 2, it can be seen that authors in this research field form a rich collaborative network. There exists a group research network with multiple associates, showing the characteristics of local concentration. Katrin Boehninggaese (32, 0.02) and Matthias Schleuing (32, 0.02) have published the most articles in this field and are also highly influential. The research topics of Katrin Boehninggaese and Matthias Schleuing include interactions between human activities and bird diversity, analysis of climate change impacts on plant and animal diversity, and spatial patterns of plant and bird diversity [45]. Moreover, the role of anthropogenic disturbance on the effect of animal seed dispersal was jointly analyzed, and many citations were obtained [46]. The collaboration of Katrin Boehninggaese and Matthias Schleuing was initiated earlier and is maintained in close collaboration. Meanwhile, close collaboration is maintained with authors such as D. Matthias Dehling (12), Eliana Cazetta, Marco A. Pizo, and Pedro Jordano, forming the largest group of authors in the field. In addition, David B. Lindenmayer has 26 publications, as the third most published author, and has an independent network of collaborations.

Teja Tscharntke (18, 0.04) is the author with the highest centrality and the highest impact in the published literature. His research focuses on the influence of landscape features on the biodiversity of plants, birds, and insects, the summary of activity patterns of birds, bats, etc., in different landscapes, and the analysis of the importance of landscape benefits generated by landscape factors on bird diversity [47,48,49,50]. The second major research network consists of authors such as Ian Macgregorfors (21) and his associated team. In addition, influential articles by authors such as Jiri Reif (10, 0.04) form a more focused collaborative network with authors such as Federico Morelli (14, 0.01).

### 3.4. Academic Development

**Figure 3:**
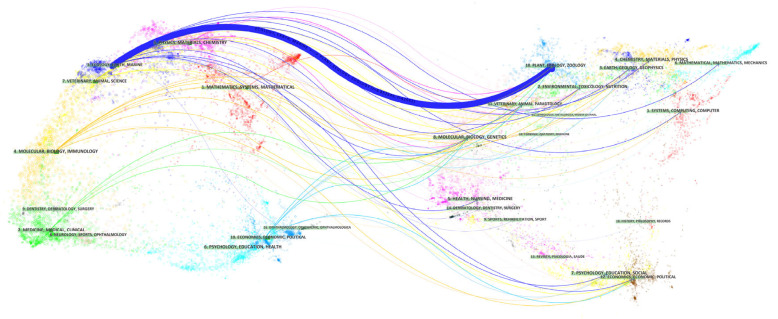
The dual-map overlay.

A dual-map overlay is an analysis method that shows the domain-level concentration of references by reference path [31]. Figure 3 was created using CiteSpace software with a source circle size = 0 and target circle size = 0. Then, the citation lines were merged by the z-score function to obtain Figure 3. The z-score function allows the highlighting of strong connections, making them easier to identify. Figure 3 shows that there is only one major citation path in this research area, and Table 3 shows this path and trends along with the names of the citation and citation regions, where the z-score is rounded to the nearest thousand. Figure 3 and Table 3 show that the field of study has evolved from ecology, earth, and marine, with a gradual shift to plant, ecology, and zoology. The literature in the fields of ecology, earth and oceans is fundamental to the field of landscape architecture and bird diversity research.

## 4. Discussion

Keywords are a strong summary of a paper’s topic, the frequency of keyword co-occurrence reflects the frontier hotspot of a research topic in a certain period, and the keyword centrality reflects the importance of keywords in the co-occurrence network. CiteSpace is used in this study to generate keyword co-occurrence mapping, keyword time zone mapping and keyword highlighting mapping for the research area. The research area is analyzed in terms of research hotspots, research history, and research preamble, respectively. In this paper, the relationship between landscape architecture and bird diversity is investigated from three aspects of landscape architecture (landscape characteristics, vegetation characteristics, and human behavioral activities) in view of the severe urbanization decline.

### 4.1. Research Hotspots

#### 4.1.1. Research Hotspots

**Figure 4:**
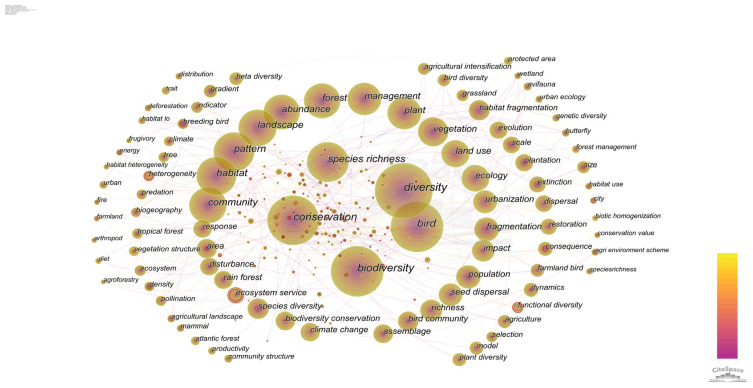
Keyword co-occurrence map.

This paper uses CiteSpace to generate keyword co-occurrence plots (Figure 4). The prune parameters (prune: pathfinder, and prune merge network) were used to create the keyword co-occurrence network graph, and the basic parameters of Figure 4 were N = 811, E = 1289, and D = 0.0039. The node size indicates the frequency of keyword occurrence; the larger the node, the more frequently the keyword occurs and the more representative of the hotspots in the domain. The depth of the connection line between nodes indicates the strength of association between nodes. Figure 4 shows that the research contents of this research domain are intertwined with each other with strong connections. Meanwhile, Table 4 counts the most frequently studied and influential keywords (the top 20) in the past two decades. Table 4 shows that the keywords with high frequency are diversity (1675), biodiversity (1338), conservation (1111), bird (1099), species richness (682), community (624), and habitat (598). The keywords show that biodiversity and conservation are widely studied by scholars in this research area. Among them, scholars focus on species richness and bird communities. Although these research keywords have been studied in high numbers, they have had less impact. Additionally, as shown in Table 4, the most influential studies in this research area are farmland (0.16), butterfly (0.14), farmland bird (0.13), biogeography (0.13), agriculture (0.11), index (0.1), consequence (0.1), and bird species richness (0.1), keywords that rarely occur, but have a profound impact on the field of study.

Therefore, according to Figure 4 and Table 4, the current research hotspots in this research area contain four research hotspots: basic research on bird communities, influencing factors related to changes in bird community characteristics, research on bird activity rhythms, and the ecological and ornamental values of birds.

First of all, the basic study of bird communities was pioneered and a solid foundation laid for this research field. The main keywords are diversity, abundance, community, pattern, habitat, bird species richness, etc., focusing on the study of bird community structure, bird behavioral characteristics and bird habitat. Moreover, the research does not only stop at the study of birds, but also gradually expands to the study of biodiversity, including butterflies and insects. Bird residence type, food habits, feeding methods and habitat layers can be used as the basis for classifying bird community structure [51]. In addition, the ecological behavior of birds can be divided into four aspects: feeding behavior, breeding behavior, spatial behavior, and community behavior. Feeding behavior is mainly the study of birds’ feeding response, including the selection of food habits and feeding bases, group feeding and feeding rhythm, etc. The feeding behavior of birds is also a behavioral adaptation to the local environment, so habitat degradation is the main reason for birds’ feeding behavior [52,53]. Birds differ in their plant preferences, and the distribution pattern of plant flowering and fruiting periods has a significant positive effect on the level of bird diversity. Bird feeding behavior is also a means for plant seed dispersal, and dispersal by birds is important for plant communities and for ecosystem stability [54,55,56]. Meanwhile, the “community structure analysis method” is used in the study of urban bird habitat landscape [57]. Moreover, it has gradually become an important indicator and hotspot for maintaining landscape ecology and sustainable urban development.

The factors influencing the changes of bird community characteristics (natural factors, human disturbance) are studied in depth. The main keywords are forest, vegetation, landscape, agriculture, farmland, tree, plant, etc. The correlation of bird diversity (abundance, multiplicity, etc.) with trees, plants, vegetation, etc., and even forests and farmland, are studied. We will investigate the correlation between bird diversity and forest and farmland and seek measures to protect and enhance them. Scholars have conducted exploratory studies on bird communities from various aspects and have achieved many useful results. There are many studies on wetland landscapes, forest landscapes, urban landscapes, and farm landscapes in different habitats [58,59,60,61]. A series of studies on the distribution types of bird habitats, the relationship between vegetation and bird communities, and the effects of forests on bird communities have been conducted in considerable depth. Among them, vegetation characteristics and structure and intensity of human disturbance are important factors affecting bird species composition. Some current domestic and international studies have shown that plant species or structures are more attractive to certain kinds of birds, that certain plant phenological periods show peaks in the degree of attraction to birds, and that factors such as tree species diameter at breast height and height, plant density, age, rotation time, tree density, plantation landscape connectivity, understory type, number of species planted, and origin of planted species largely determine local bird diversity and also affect local bird diversity [62]. For example, native plant vegetation is more capable of enhancing bird diversity [63]. The positive benefits of tree cover density should be very important [64]. There is a significant linear positive effect of shrub density on both bird species richness and abundance [65]. Tree and shrub density were also the main drivers of bird community composition, followed by tree species diversity and landscape forest cover. The degree of variation in bird diversity varies more with mean breast size. Compared to other arthropods and mammals, birds are more sensitive to disturbance by human activities. This is especially true for the conversion of forests to agriculture. Although several studies have investigated the effects of disturbance on the functional diversity of birds, the results varied widely, with differences between positive, negative and no effects [62,66]. It is possible that this variation is due to the type of disturbance investigated in the study, such as land use change and logging, as well as the intensity or frequency and the wide variation in environmental type [67,68].

Focusing on the study of the rhythm of bird activity, the key words are these: conservation, land use, urbanization, impact, management, population, consequence, conservation planning, etc. With the advancement of urbanization, climate change, and land use change, the change of bird community characteristics is gradually emphasized as an important ecological indicator into cities. In parallel with the development, landscape pollution has increased, which has a greater impact on birds. Therefore, more and more studies are focusing on the rhythm of bird activity, seeking strategies to address the decline in environmental quality and biodiversity. Bird community characteristics change with temporal, spatial and temporal changes. As urbanization accelerates, natural forests are gradually replaced by gray buildings and artificial forests, leaving less and less space for birds to roost. Urban green spaces such as parks, gardens, campuses and greenways have become important refuges for urban biodiversity, even playing an equal or greater role than nonurban environments [69]. Due to the rise of urban parks, urban landscape diversification is built upon. The urbanization process has a certain species conservation function, bird communities change in the same direction as plant communities, and the spatial and temporal changes in bird communities are inextricably linked to the food provided in urban forests and to habitat characteristics [70]. Research on the spatial variation of bird community characteristics is broadly divided into two aspects. On the one hand, there are large scale studies, which usually study different patch sizes [71], variation in patch shape [72], land use type, elevation, temperature, climate [73], etc. On the one hand, microscale studies were conducted to examine the differences in vegetation landscape, water landscape, management frequency, human disturbance, noise, and other factors between different site types within a single park in relation to changes in bird community characteristics for linkage and analysis. For example, current and past climate, elevation, green space, normalized vegetation index (NDVI), and population density [74]. Broadly, the composition of bird communities varies with the gradient of urbanization [75]. In addition, species richness decreases with increasing urbanization and bird richness and density increases with increasing urbanization. How different land use plots such as protected forests, forest sanitation areas, and urban farmland affect the behavior of birds such as foraging, roosting, and breeding has been examined, and the characteristics of their changes throughout the year were studies [76]. The correlation of bird diversity with urban green space was noted in 112 urban green spaces in 51 cities of eight countries [77]. Therefore, bird diversity is often used as an important assessment indicator of the strengths and weaknesses of environmental ecology. In landscape construction, urban construction, and artificial forestation, bird diversity is often used as the main goal of landscape construction to be close to nature. Therefore, landscape construction and management methods with bird diversity as the main goal have been proposed and practiced in research. 

Last but not least, bird habitat construction was born, and the ecological and ornamental values of birds are becoming better known to the public. Urban birds may play a key role in providing various aspects of the landscape and ecology. One of the greatest values and roles of urban birds is to serve as a link between the natural environment and the increasingly deprived urban population [78]. Birds in urban residential areas are often valued for their color and song, for providing mental and physical health, as indicators of seasonal change, education, and familiarity for residents [79]. Birds are also a source of inspiration for arts and recreational activities such as bird watching and wildlife gardening [80]. In addition, birdwatching tourism is also a low-carbon tourism mode, and most of the current studies focus on the study of site design of birdwatching locations and the planning of birdwatching routes. However, most of them only stay in the planning of one bird watching route, without diversified design, and lack of specifics regarding bird watching as to the type, location and time, one by one correspondence, and low practicability. There is a lack of targeted design for specific groups of people such as the elderly, children, and birdwatching enthusiasts [81]. At the same time, the design of bird watching routes is based on “bird song”, and there are few designs that advocate barrier-free bird watching [82].

#### 4.1.2. Research History

**Figure 5:**
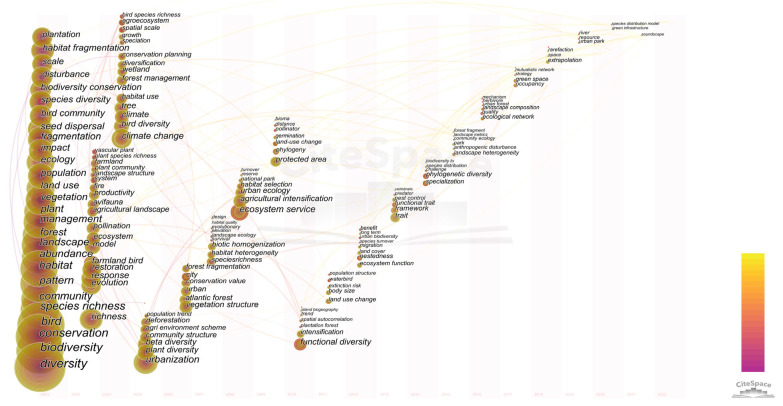
Research keyword time zone view map.

**Figure 6:**
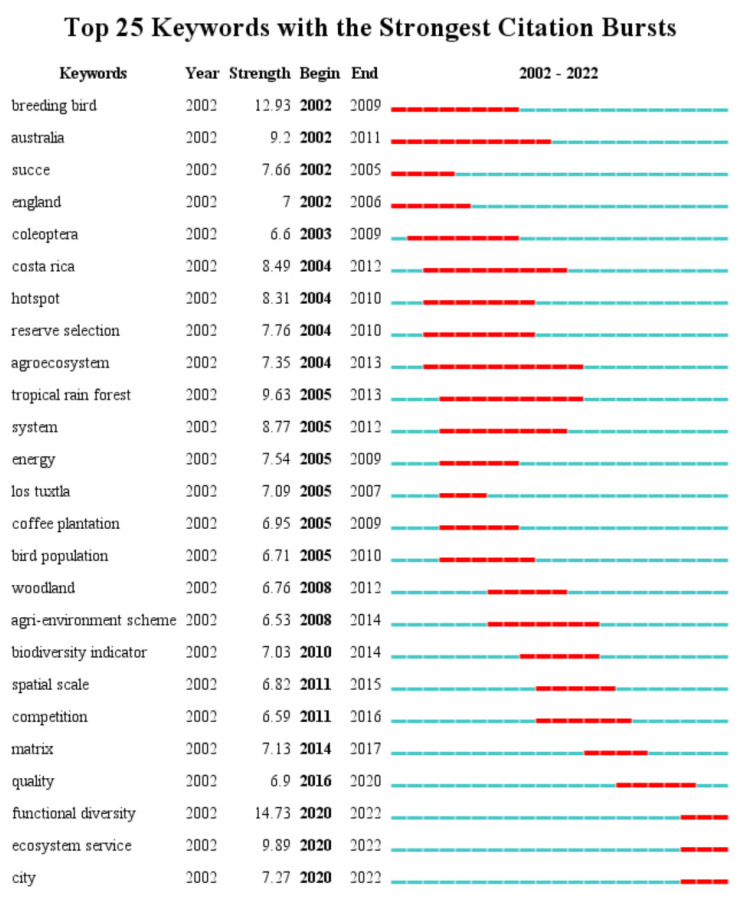
Top 25 keywords with the strongest citation bursts.

The time zone map provides a clear picture of the development of the research field. In this paper, CiteSpace was used to obtain the time zone map (Figure 5), using prune: pathfinder, and prune merge network, with the rest of the parameters as default parameters. Figure 5 shows the richness and diversity of the development process of this research area. At the same time, the study is constantly evolving in the development process, generating new keywords every year. Figure 6 shows the history of the research field through a total of four developmental stages.

Phase I (2002–2004): The key words generated in 2002 had a profound influence on future research and were practiced in the process of research development. The fundamental studies of bird communities, including bird habitat surveys, influencing factors related to changes in bird community characteristics, and ecological values of birds, all started in this period. The basic research on bird communities includes the study of basic diversity, abundance, habitat, pattern, species richness, and community. In addition, the ecological value of birds has been studied. In addition, the research shows that the diversity of birds decreases with development. Therefore, it was realized that scientific and effective management measures can have a positive effect on bird diversity.

Phase II (2005–2009): Studies related to the influencing factors of changes in bird community characteristics, the rhythm of bird activities, and the ecological value of birds were emphasized in this phase. The forest environment changed with climate change, urbanization development, and the rise of agroforestry industry. These land use changes occupy bird habitats. This leads to urban ecological decline, habitat homogenization, and a declining quality of vegetation structure. The bird community structure is unstable, the number of species decreases, and diversity decreases, and even the ecosystem services weaken. Society became aware of the ecological value of bird diversity conservation. In addition, the sites of concern at this stage begin to diversify to include water, the countryside, and national parks.

Phase III (2010–2015): In this phase, the research on the ecological value, function and role of birds was further promoted, focusing on the functional diversity and functional traits of birds, landscape, and environmental factors, while the investigation of bird communities themselves was continued and extended to insects, etc. The study of the factors influencing the change in bird community characteristics was further developed. In addition, the important role of landscape composition of urban biodiversity and landscape heterogeneity on urban bird diversity at this stage was realized, among which the effective transformation of land use is more critical. As a result, parks and green areas have been consciously built in cities to restore urban bird diversity. However, the conservation and enhancement of urban bird diversity takes a long time to operate. The survival and diversity of urban birds are still facing great challenges.

Phase IV (2016–2022): This phase focuses on the impact of macrostructure on bird diversity. With the urban expansion, the ecological quality of the city is weakened and the bird diversity is reduced. Thus, it is proposed to value urban planning and environmental heterogeneity and construct ecological networks scientifically. In particular, the focus is on urban landscape construction and ecological construction through strategies such as tree plantation, rational creation of green space, urban green space, urban park, and green infrastructure. In addition, factors influencing changes in bird community characteristics, such as increased elevation and tallgrass prairie were studied.

#### 4.1.3. Research Frontiers

Keyword burst detection is used to detect the decline or rise of a keyword, and the greater the burst intensity the more frequently the keyword appears in a given period, indicating that many studies related to it have appeared in that period. These keywords are called burst keywords. These burst keywords can help scholars to identify new trends in that research area. In this paper, CiteSpace was used to obtain the top 25 keywords in this research area in terms of explosiveness (Figure 6), and all parameters are the default parameters of the system. In this paper, the description of the highlighted words will be developed in chronological order.

Early outbreak vocabulary: breeding bird (12.93), Australia (9.2), success (7.66), England (7), Costa Rica (8.49), hotspot (8.31), reserve selection (7.76) and agroecosystem (7.35). These keywords indicate that, during this period, the main goal of this research area was to improve breeding bird diversity [83]. The basic research on bird communities and the correlation of factors influencing the changes of bird community characteristics were actively carried out. In the second half of the twentieth century, countries such as Australia, England, etc., underwent modernized industrial agricultural production. However, the environment was damaged, and biodiversity was lost. Therefore, different countries adopted different environmental policies to enhance biodiversity, and bird diversity and bird habitat became a hot topic of research. In particular, bird reserve selection and the breeding of urban birds are important frontiers in this research area. 

Medium-term outbreak vocabulary: tropical rain forest (9.63), system (8.77), Los Tuxtla (7.09), coffee plantation (6.95), bird population (6.71), woodland (6.76), agri-environment scheme (6.53), biodiversity indicator (7.03), competition (6.59). The ecological value of birds, the factors influencing the changes in the characteristics of bird communities, and the activity patterns of birds continue to be studied at this stage. However, the scope of research was further expanded, not only to single habitats and vegetation structures, but also to systematic and agro-ecological systems. In addition, more macroscopic plans and strategies, such as agro-environmental plans and biodiversity indicators, are being developed. In addition, the large-scale deforestation of tropical forests worldwide has become a major driver of biodiversity loss and decline [84].

Recent outbreaks of vocabulary: city (7.27), matrix (7.13), functional diversity (14.73), ecosystem service (9.89). Recently, rapid urban expansion has led to the destruction of habitats for wildlife, including birds. Urban ecosystems are in urgent need of restoration and a series of studies on biodiversity, including bird diversity, are of great interest. Urban green spaces, including urban parks, are important habitats for birds, and therefore an increase in the functional diversity (size, heterogeneity, etc.) of urban green spaces has a positive impact on bird diversity. In addition, the research frontiers in this phase pay more attention to the construction of urban networks and the linking of urban green spaces. Incorporating different landscape matrices into urban planning can maintain the diversity of urban birds. Moreover, it is a more cost-effective solution [85,86].

In summary, the research in this research area is progressive and closely follows the social development situation. The research area has progressed from small to large, from surface to deep. From focusing on the basic research of bird communities, it has developed to research on the influencing factors (natural factors, human disturbances) related to the changes in bird community characteristics. We even push the research in different dimensions of time and space. Additionally, in this way, the theoretical and action plans in urban planning practice are discussed in reverse.

### 4.2. The Relationship between Landscape Construction and Bird Diversity

Urban biodiversity has declined rapidly as a result of rapid urban expansion. Urban landscape factors are important in determining both the survival and maintenance of bird species diversity [87,88]. It is important to investigate the relationship between landscape creation and urban bird diversity, so this collection focuses on the analysis and discussion of urban landscape creation and bird diversity. Bird diversity in urban areas depends mainly on landscape characteristics (fragment area, isolation, shape, habitat diversity, etc.), vegetation characteristics (plant species, plant phenology, tree size, plant density, plant structure, etc.), and human behavioral activities (noise level, number of people). In particular, landscape characteristics of land structure use and vegetation structure are the best predictors of taxonomic diversity, functional diversity and evolutionary uniqueness of bird communities in urban parks [89]. Moreover, human behavioral activities still have some influence on birds. It so happens that these influences are part of the urban landscape and can be addressed through scientifically based landscaping to address the declining diversity of birds. In addition, the factors influencing bird diversity vary greatly from region to region and from site to site, and local studies are needed to develop appropriate conservation plans [70]. Therefore, this paper focuses on the relationship between landscape architecture and bird diversity from three aspects of landscape architecture (landscape characteristics, vegetation characteristics, and human behavioral activities).

#### 4.2.1. The Relationship between Landscape Features and Bird Diversity

Urban landscapes are rich in types, including urban parks, street green spaces, botanical gardens, etc. These urban landscapes include different types of landscapes such as urban parks, urban street green spaces, large wetlands, rain gardens, etc. Moreover, there may even be a special green space wetland in the city, and wetland creation is a common conservation measure to mitigate the loss of biodiversity caused by global wetland destruction. Studies have shown that several small (1 ha) wetlands with high flooded areas are better able to nurture wetland bird communities [58]. Spatial heterogeneity of the landscape is a key factor affecting biodiversity [90]. The construction of different landscape features such as landscape structure, habitat configuration, spatial arrangement, area, isolation, shape index, distance to the city center and habitat diversity can enhance landscape spatial heterogeneity, and thus influence bird richness. The most useful indicators of landscape heterogeneity for bird diversity are land cover richness, weighted edge density and edge density as a scale [91]. In addition, the distance of the site from the main road and water bodies, the hardness rate of the site, and the openness of the site will also have some influence on bird diversity [92,93]. For example, even in forests with low levels of recreation, highly sensitive species (large FID) tend to avoid areas near trails. The shift from natural habitats to human-dominated landscapes has had a significant impact on bird communities in the study area. Human-dominated habitats inhabited more bird species worthy of conservation [94]. Therefore, urban plans should include measures to provide suitable breeding habitats for threatened forest species to increase their populations, thereby increasing biodiversity and promoting the well-being of urban residents [60]. Urban planning in combination with green spaces can contribute to the diversity of birds in cities [3].

#### 4.2.2. The Relationship between Vegetation Characteristics and Bird Diversity

Certainly, more green spaces and more plant species promote higher bird diversity. Plant landscape camping in green space, and features such as plant species, plant dominant family, plant canopy characteristics, plant density and cover, canopy width, diameter at breast height, height, number, density, etc., have an effect. First, the selection of plant species, including food source species, deciduous species, coniferous species, etc., is important, as are measures to attract urban birds through the selection and matching of greening tree species [95]. Some birds are particularly adapted to deciduous forests, and the physical advantages of deciduous forests are crucial to their distribution, such as the medium-spotted woodpecker, which specializes in deciduous forest food and breeding resources [96,97]. Overall bird diversity in coniferous stands is low [98]. Moreover, the effect of plant canopy structure variables on predicting bird diversity associated with coniferous forest habitats was the most important [99]. Unmanaged, large, and dense urban forests can serve as a safe habitat for large numbers of birds, regardless of the level of disturbance in the urban environment [100]. For the average percentage of canopy cover, ground (below 1 m), shrub level (1 to 5 m), low canopy (5 m to canopy level) and top level, each life form percentage is calculated independently so that total cover can be over 100% [101]. Vegetation cover under the forest canopy is key to increasing species diversity and richness [102]. The current stage uses LiDAR to measure and calculate the relationship between plant canopy features and bird communities [103]. The effect of canopy density on bird diversity is generally bell-shaped, while the effect on functional diversity and phylogenetic diversity is U-shaped [104]. Meanwhile, plant density and cover usually contribute to increase bird species richness and diversity. Tree and shrub density were the main drivers of bird community composition, and shrub density had a significant linear positive effect on both bird species richness and abundance [65]. This is followed by tree species diversity and landscape forest cover [105]. Tree cover in oases and olive groves positively affects bird diversity, and the positive benefits of tree cover density should be important when interpreting bird diversity in oases and olive groves [64]. In addition, the degree of variation in bird diversity varied more with mean breast size. Benjamin James Barth et al. found that the number of species and total bird abundance were positively correlated with the total number of mature trees retained within the vegetated street [106].

#### 4.2.3. The Relationship between Human Behavioral Activities and Bird Diversity

The impact of various anthropogenic disturbances (e.g., agriculture, logging, urbanization, etc.), coupled with increasing population demand, is a constant threat to biodiversity on a global scale. The consequences of human pressure are not limited to species loss, but also affect other biodiversity aspects such as phylogenetic, genetic, and functional diversity. In the field of bird biology, reduced bird community activity is associated with reduced abundance and reduced species richness [107]. When exposed to human presence, birds undergo significant changes in behavior and physiology and respond immediately, with reactions such as increased vigilance, flight, and the release of stress hormones, which in turn may have effects on individual bird health and bird population dynamics. In addition to these direct effects, indirect effects can also affect wildlife primarily through the loss or alteration of bird habitats. The response of wildlife to human recreational activities depends not only on the characteristics of the animals involved (e.g., species, sex) and the type of human disturbance (e.g., noise level, number of people), but also on environmental conditions (e.g., habitat) and animal ecotype [108]. The temporal variation of disturbance events also shows different changes in bird communities, a phenomenon that also requires more attention from researchers [109]. For the study of human disturbance, the startle distance is one of the most frequently studied indicators, and the startle distance of different species of birds responding to human approach varies, with a high degree of similarity between birds of different species of the same family. For example, the startle distances of finches are about 16.2 m, those of chickadees are about 42.9 m, and those of falcons are up to 134.5 m [110]. Environmental noise is a limiting factor for urban birds, and noise level is a key influence on whether migratory birds call. Although the structure of vegetation features has a more significant impact on bird communities, influences such as human disturbance, noise level, and frequency of site management cannot be ignored [93,111].

Anthropogenic behavior: Studies have shown that trails and roads can significantly affect bird community composition and abundance, not only by altering habitats along trails, but also primarily through recreationist use. There were significant differences in bird startle distances when humans approached birds between park and cemetery-dwelling individuals, and differences in human activity villages between the two habitats made differences in startle distances [112].

Site noise: Anthropogenic noise is becoming more prevalent in the world and has been shown to affect many animal species, including birds. The effects of such noise were measured in a neotropical urban park to assess how noise affects bird diversity and species richness. Noise was negatively correlated with bird species richness, total abundance, and pellet-feeding species richness [113].

Frequency of site management: Bird responses varied by land use and season, and important and beneficial behaviors were observed in sample plots with high site management frequency. Birds showed different activities in different sample sites [114].

## 5. Conclusions

Rapid urban development poses a global challenge to mitigate the impacts of urbanization on biodiversity, and enhancing and conserving urban bird diversity is an important component of biodiversity enhancement. Landscape architecture is one of the indispensable technical tools. In this study, a bibliometric study was conducted by mining the literature studying landscape architecture and bird diversity to discover the background knowledge and research frontiers in this research area. Therefore, analysis using CiteSpace can help scholars to quickly understand a field of research. Firstly, this research area has a research history with an increasing number of publications in the literature year by year. Secondly, most countries and regions around the world are involved in this research area. Moreover, the authors are highly involved and there is a research network of authors associated with multiple people, showing the characteristics of local concentration. At the same time, the discipline maintains its traditional development during the development process. In addition, this study delves into four research hotspots (fundamental studies of bird communities, factors influencing changes in bird community characteristics, studies of bird activity rhythms, and ecological and ornamental values of birds), four developmental stages (2002–2004, 2005–2009, 2010–2015, 2016–2022), and several research frontiers (breeding birds, tropical rain forest, functional diversity, etc.). Meanwhile, the relationship that exists between landscape architecture and bird diversity is discussed in three aspects (landscape characteristics, vegetation characteristics, and human behavioral activities). This paper systematically summarizes the knowledge of the literature in this field to deepen the understanding of this research area and to quickly grasp the correlation between landscape architecture and bird diversity. The results of this study will help to reveal the important role of scientific landscape architecture in achieving the mitigation of urban bird diversity decline.

In the future, there is a need for landscape construction around the goal of bird diversity. Firstly, we will use multiple big data to monitor conservation and development dynamics, and effectively summarize the behavioral characteristics and spatio-temporal activity patterns of birds. We will draw a cloud map of bird distribution in each block combined with GIS to obtain dynamic big data and grasp the changes in bird diversity. Secondly, we will reasonably consider the activity characteristics of birds when constructing the landscape, and thoroughly study the landscape construction strategy and management principle of harmonious coexistence of human and birds, in addition paying attention to the landscape features, the construction mode and level of landscape vegetation, and the need to consider human behavior activities. Plots with good ecological substrates will be used as bird diversity enhancement areas, while the opposite will be focused on the space for human activities. We also will pay attention to the transition of levels, and reasonably reserve some plots for harmonious coexistence between humans and birds. At the same time, appropriate science education should be carried out to get into birds, ecology, and nature. Finally, multi-disciplinary and multi-departmental collaboration will establish a construction model that is both scientific and practical. It is often not enough to focus on a single block to enhance bird diversity, but rather necessary to link it to urban planning to form an overall block chain. This often requires multidisciplinary integration and innovative construction and planning models. This will help to mitigate the decline of bird diversity, preserve biodiversity, and enhance human well-being.

## Figures and Tables

**Table 1:** The 20 countries or regions with the highest total number and centrality of research papers published in the study.

Rank	Countries	Count	Centrality	Year	Countries	Count	Centrality	Year
1	USA	1311	0.08	2002	England	488	0.28	2002
2	England	488	0.28	2002	Australia	387	0.1	2002
3	Germany	410	0.06	2002	France	253	0.1	2002
4	Australia	387	0.1	2002	USA	1311	0.08	2002
5	Spain	306	0.03	2002	Belgium	76	0.08	2004
6	Peoples R China	290	0.01	2003	Germany	410	0.06	2002
7	Brazil	285	0.03	2002	Cameroon	13	0.06	2005
8	Canada	261	0.03	2002	Portugal	102	0.05	2003
9	France	253	0.1	2002	Scotland	96	0.05	2002
10	Switzerland	172	0.02	2002	South Africa	158	0.04	2002

**Table 2:** The 10 authors with the highest total number and concentration of research papers published in the study.

Rank	Authors	Count	Centrality	Year	Authors	Count	Centrality	Year
1	Matthias Schleuning	32	0.02	2012	Teja Tscharntke	18	0.04	2008
2	Katrin Boehninggaese	32	0.02	2008	Jiri Reif	10	0.04	2015
3	David B Lindenmayer	26	0.00	2009	Petr Pysek	4	0.04	2017
4	Ian Macgregorfors	21	0.01	2010	Holger Kreft	4	0.04	2017
5	David P Edwards	20	0.01	2013	Matthias Schleuning	32	0.02	2012
6	Teja Tscharntke	18	0.04	2008	Katrin Boehninggaese	32	0.02	2008
7	Federico Morelli	14	0.01	2015	Yann Clough	8	0.02	2009
8	Nina Farwig	12	0.01	2008	Frederic Jiguet	6	0.02	2007
9	D Matthias Dehling	12	0.01	2014	Nico Bluethgen	4	0.02	2016
10	John O’halloran	11	0.00	2010	Ben Collen	4	0.02	2014

**Table 3:** The key citation trends.

Citing Region	Cited Region	z-Score
Ecology, earth, marine	Plant, ecology, zoology	8.853

**Table 4:** The 20 keywords with the highest total number and concentration of research papers published in the study.

Rank	Keywords	Count	Centrality	Year	Keywords	Count	Centrality	Year
1	Diversity	1675	0.01	2002	Farmland	57	0.16	2003
2	Biodiversity	1338	0	2002	Butterfly	76	0.14	2002
3	Conservation	1111	0	2002	Farmland bird	135	0.13	2003
4	Bird	1099	0.01	2002	Biogeography	101	0.13	2002
5	Species richness	682	0	2002	Agriculture	122	0.11	2002
6	Community	624	0.01	2002	Index	10	0.1	2018
7	Habitat	598	0	2002	Consequence	136	0.1	2002
8	Pattern	588	0.01	2002	Bird species richness	33	0.1	2004
9	Landscape	520	0	2002	Rain forest	190	0.09	2002
10	Abundance	495	0	2002	Environment	28	0.09	2003
11	Forest	444	0	2002	Competition	34	0.09	2002
12	Management	420	0.01	2002	Hotspot	40	0.08	2002
13	Plant	416	0	2002	Atlantic forest	87	0.08	2006
14	Vegetation	349	0.01	2002	Vertebrate	8	0.07	2013
15	Land use	346	0.05	2002	Tree	110	0.07	2004
16	Ecology	294	0	2002	History	24	0.07	2003
17	Population	271	0.01	2002	Evolution	168	0.07	2003
18	Impact	264	0.05	2002	Diversification	53	0.07	2004
19	Urbanization	262	0.02	2005	Diet	56	0.07	2002
20	Fragmentation	252	0	2002	Conservation planning	50	0.07	2004

## Data Availability

The data used to support the findings of this study are available from the corresponding author upon request.
